# Anatomical study of the sternoclavicular joint using high-frequency ultrasound

**DOI:** 10.1186/s13244-022-01167-x

**Published:** 2022-04-05

**Authors:** Timothée Olivier, Kevin Kasprzak, Matthias Herteleer, Xavier Demondion, Thibaut Jacques, Anne Cotten

**Affiliations:** 1 Service de Radiologie et Imagerie Musculosquelettique, CCIAL, CHU Lille, 59037 Lille, France; 2 Laboratoire d’Anatomie, Faculté de Médecine, Univ. Lille, Lille, France; 3 Unité de Taphonomie Médico-Légale et Anatomie (UTML & A), EA 7367, Univ. Lille, Lille, France; 4 Faculté de Médecine, Univ. Lille, Lille, France; 5 MABLab – Marrow Adiposity and Bone Lab ULR4490-Univ. Lille, Lille, France

**Keywords:** Anatomy, Articular ligaments, Shoulder, Sternoclavicular joint, Ultrasound

## Abstract

**Objectives:**

The purpose of the present study was to determine whether ultrasound enables assessment of sternoclavicular structures.

**Methods:**

A preliminary study in 3 cadavers was followed by an ultrasound study, performed by 2 musculoskeletal radiologists working in consensus, in 59 patients without history of trauma, surgery or pain in the sternoclavicular joint. The visibility, echogenicity and thickness of the sternoclavicular structures were assessed.

**Results:**

The anterior sternoclavicular ligament and the interclavicular ligament could be seen in all patients (mean thickness: 1.4 mm and 1.3 mm, respectively). The articular disc was clearly seen in 66.1% of cases, and shoulder antepulsion enabled analysis in an additional 20.3%. Intra-articular joint gas was frequent (33.89% of cases), preventing analysis of the disc in 2 patients. Only the superficial anterior aspect of the clavicular and sternal articular cartilages could be assessed. Joint effusion was seen in 6.8% of cases. Clavicular osteophytes, sternal osteophytes and bone irregularities at the anterior sternoclavicular ligament insertion were detected in 33.9%, 16.9% and 16.9% of cases, respectively.

**Conclusion:**

The anterior sternoclavicular ligament, interclavicular ligament and anterior intra-articular structures can be visualized by ultrasound. This means of assessment may have clinical applications, particularly in patients with trauma or microtrauma.

## Key points

- Most of the anterior sternoclavicular structures can be visualized by means of ultrasound.
- Antepulsion of the shoulder may improve the analysis of the intraarticular disc.
- Ultrasound assessment of the sternoclavicular joint might be useful in patients with trauma or microtrauma.

## Introduction

The sternoclavicular joint is a synovial saddle joint that connects the sternum with the clavicles. It is the only true joint which connects the appendicular skeleton of the upper limb with the axial skeleton of the trunk [1]. The sternoclavicular joint is solicited in each movement of the upper limb, and this joint is the key to perform the upper limb full range of movement [1]. Because only half of the medial clavicle articulates with the sternum, the stability of the joint also relies on soft tissue and ligamentous attachments.

The assessment of the anatomy of the sternoclavicular joint using imaging mainly relies on CT and MRI [2–5]. Several studies reported the contribution of ultrasound in detecting sternoclavicular synovitis [6–9] or guiding joint infiltration [10] but to the best of our knowledge, no descriptive study of the anatomy of the sternoclavicular joint by means of high-frequency ultrasound has been reported in the literature. This is all the more surprising as this joint is superficial and therefore easily accessible to this imaging modality. Knowledge of the normal appearance of the intra-articular structures (intra-articular disc, cartilage) and stabilizing ligaments might be useful for detecting minor changes of this joint, including trauma and microtrauma, which are not a usual indication for ultrasound assessment of the joint.

The purpose of the present study was to determine normal sternoclavicular joint anatomy using ultrasound.

## Materials and methods

### Anatomical cadaver study

The initial study was undertaken on three male cadavers in order to gain a better understanding of the anatomy of the sternoclavicular joint. The cadavers (75, 78 and 80 years of age) were donated for the purposes of research and education on human anatomy. They did not present any history of injury or surgery in the dissected area. Embalming was carried out using a preparation including distilled water, glycerin, methanol and phenol, conserving tissue consistency and joint range of motion. Dissections were performed by a trained anatomist.

In one cadaver, skin and subcutaneous tissue of the lower neck and upper part of the torso were removed, exposing the sternocleidomastoid and the pectoralis major muscles. Resection of the sternal and clavicular attachments of these muscles, which were later reflected, allowed bilateral exposure of the underlying sternoclavicular joints, subclavius muscles and clavipectoral fasciae. Careful dissection was undertaken to identify the anterior sternoclavicular, interclavicular and costoclavicular ligaments. The capsule of each sternoclavicular joint was then cut open to reveal the articular disc.

Then, the other two cadavers were used to confirm the accuracy of the ultrasonographic depiction of the sternoclavicular joint and its surrounding components. This examination was performed using a 24 MHz ultrahigh-frequency linear probe (Aplio i800 ultrasound device, Canon Medical Systems).

The target ligaments and the tendons of the sternocleidomastoid muscles were transfixed by two musculoskeletal radiologists (a senior musculoskeletal radiologist and a radiology resident), consensually, under continuous ultrasound control, using one Ethicon Vicryl® 5/0 stitch with 19 mm reverse cutting needle. Each sternoclavicular joint was then dissected as described above, to ensure that the ultrasound target corresponded to the anatomical target.

### Patients and ultrasound routine

Between September 2019 and June 2020, 59 patients (20 females and 39 males, with a median age of 35 years) were included in this study conducted in the Musculoskeletal Imaging Department of the University Hospital of Lille, France. Inclusion criteria comprised referral to our department for clinical suspicion of shoulder trauma without radiographic fracture, routinely assessed using ultrasound in our department, with asymptomatic ipsilateral sternoclavicular joint. This sternoclavicular joint was used for the purposes of our study and was assessed consensually by the same two radiologists as in the cadaver study. Exclusion criteria comprised age under 18 years, history of trauma, surgery or pain in the ipsilateral sternoclavicular joint, and incomplete ultrasound examination. All patients provided informed consent. This study was declared to and approved by the institutional review board (CRM-1906–009).

All the ultrasound examinations were used a 24 MHz ultrahigh-frequency linear probe (Aplio i800 ultrasound device, Canon Medical Systems); an ultrahigh-frequency 22 MHz hockey stick probe was also used when wavy outlines of the superior chest wall (e.g., jugular notch or overhang of the clavicles) made analysis of the anatomical structures difficult. An additional gel pad was also used to overcome this difficulty.

Fifty-nine sternoclavicular joints (31 right, 28 left) were analyzed. Patients were examined in supine position, arm alongside the body, elbow flexed and the palm of the hand on the umbilicus. They were asked to perform an antepulsion of the shoulder if intra-articular assessment of the sternoclavicular joint was not optimal.

Several qualitative and quantitative parameters were assessed. The visibility of the interclavicular, costoclavicular and anterior sternoclavicular ligaments and articular disc was classified as good or poor. Echogenicity and thickness were assessed. For the interclavicular ligament, proximity to the upper part of the manubrium sterni was assessed.

The anterior clavicular and sternal bone surfaces adjacent to the joint were also assessed for osteophytes (joint-surface margin ossification extending the joint) and for irregularities of the bone surface at the capsuloligamentous attachments (defect or bone formation of the cortical line). Regarding intra-articular structures, the thickness of the sternal and clavicular cartilages was measured at their deepest analyzable part. Joint width was measured between the deepest analyzable bone margins. The articular disc was studied. The joint space was assessed for joint effusion and gas content. Finally, power Doppler was used in each examination to detect synovial vascularity.

## Results

### Anatomical study on cadavers

**Fig. 1:**
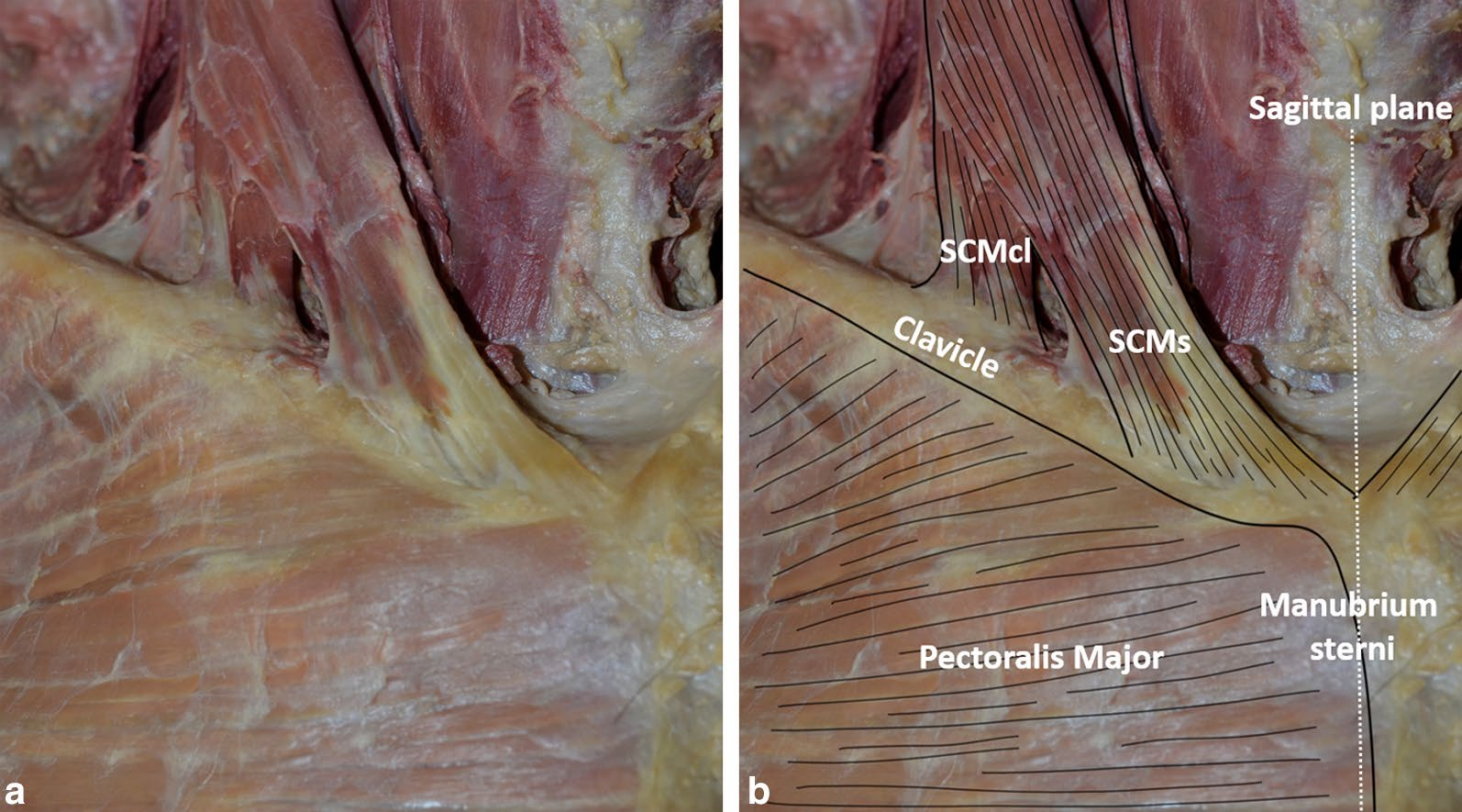
Anterior view of the right base of the neck and upper torso after removal of skin and subcutaneous tissue without (**a**) and with (**b**) annotations. SCMs: sternal tendon of the sternocleidomastoid muscle; SCMcl: clavicular tendon of the sternocleidomastoid muscle

**Fig. 2:**
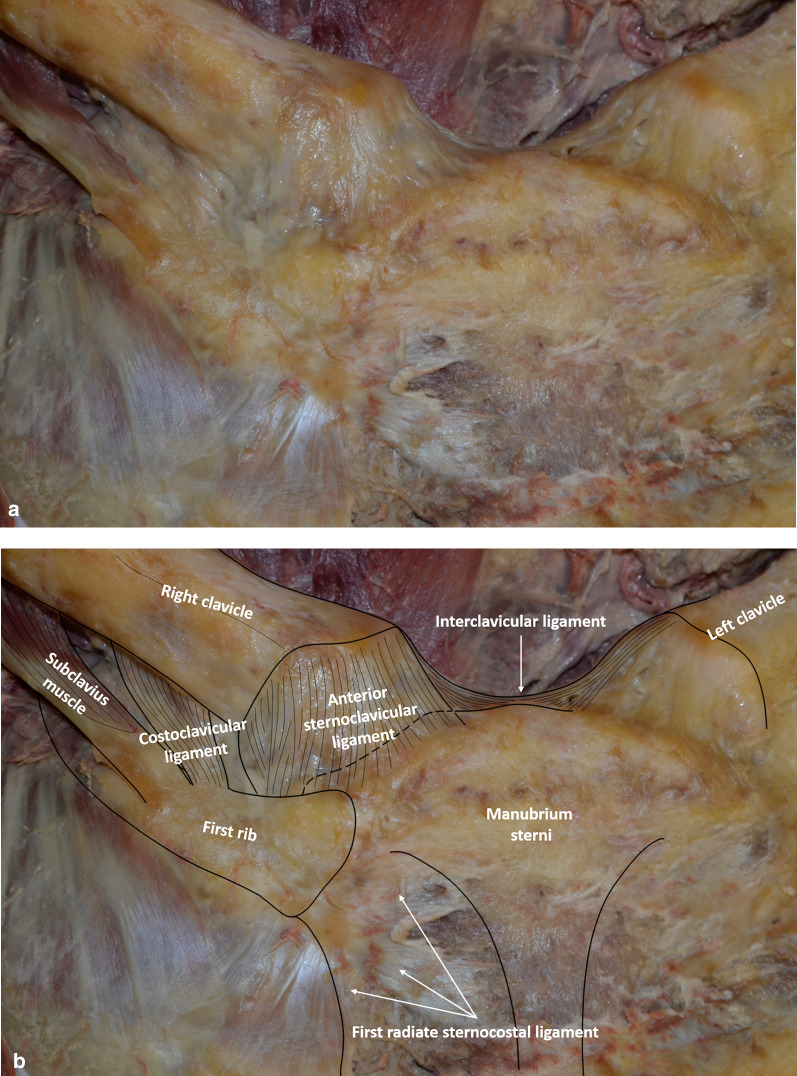

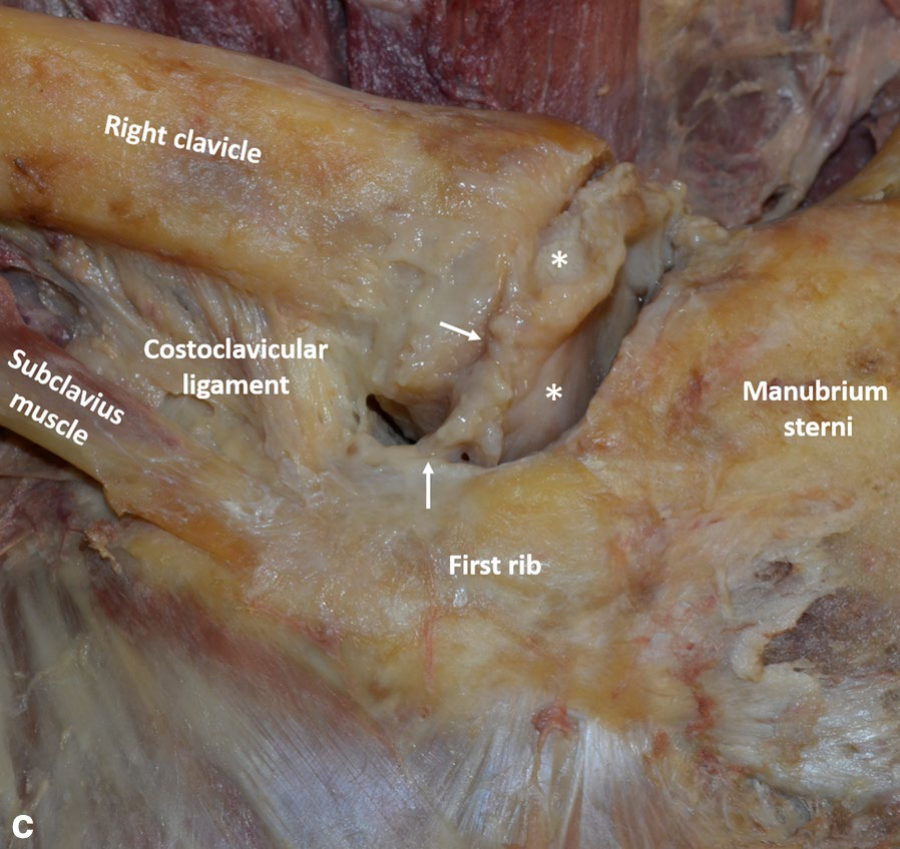
Anterior view of the right sternoclavicular joint without (**a**) and with (**b**) annotations. The sternocleidomastoid tendons and the pectoralis major muscle have been removed, exposing the anterior sternoclavicular ligament, interclavicular ligament and costoclavicular ligament. **c** Anterior view of the right intra-articular disc (*) after removal of the anterior sternoclavicular capsuloligamentous structures. Note persistent anterior capsuloligamentous fibers inserted on both the disc and the first costal cartilage (small arrows)

Removal of skin and subcutaneous tissue of the lower neck and upper part of the torso exposed the sternocleidomastoid tendons and pectoralis major muscle in front of the sternoclavicular joint (Fig. 1). After resection of the muscle, the anterior sternoclavicular ligament was seen as a broad structure covering most of the anterior surface of the sternoclavicular capsule (Fig. 2). It attached below on the upper and anterior part of the manubrium sterni, surrounding the anterior edge of the clavicular notch. Its most lateral, nearly vertical, fibers inserted on the costal cartilage of the first rib. Above, it attached on the anterior and superior aspect of the sternal end of the clavicle.

The interclavicular ligament consisted of fibers coursing between the right and left clavicles, from one sternal end to the other (Fig. 2). Fibers were seen attached to the jugular notch, creating a superior sternoclavicular ligament.

The costoclavicular ligament formed a thick structure, rhomboid in shape. It inserted on the upper aspect of the first costal cartilage (Fig. 2). It coursed upward and laterally to insert on the medial quarter of the inferior aspect of the clavicle.

In two cadavers (4 joints), the articular disc divided the sternoclavicular joint in two distinct spaces (Fig. 2). It attached to the upper and posterior aspect of the sternal end of the clavicle and, by its circumference, to the sternoclavicular capsule and the anterior and posterior sternoclavicular ligaments. The articular discs of the third cadaver were indistinguishable from the joint surfaces, due to marked degenerative changes.

Dissection of the second and third cadavers confirmed that the sternocleidomastoid tendons, anterior sternoclavicular ligament, interclavicular ligament and articular disc were correctly transfixed using ultrasound. Due to their close relationship, the sternal tendon of the sternocleidomastoid muscle and the anterior sternoclavicular ligament were frequently transfixed together. The costoclavicular ligament was not transfixed, as it could not be correctly identified by ultrasound.

### Ultrasonographic study in patients

**Fig. 3:**
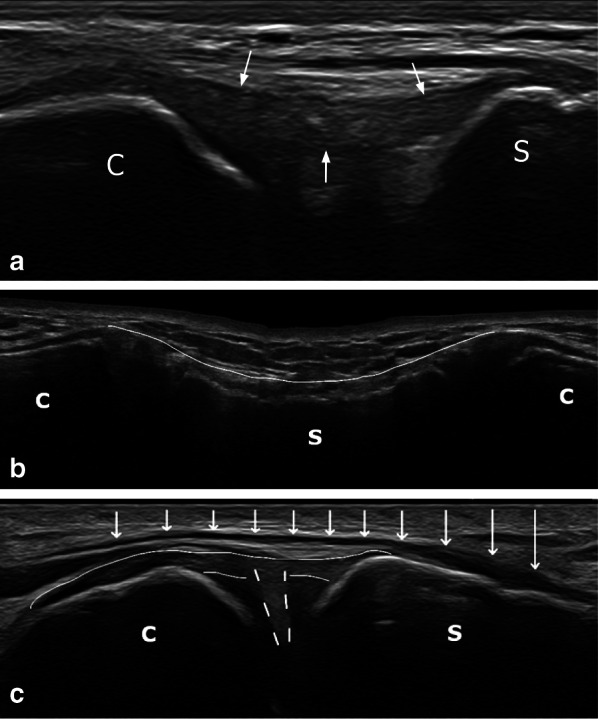
Ligaments and tendons of the sternoclavicular region. **a** Anterior sternoclavicular ligament (white arrows) (upward and outward oblique transverse plane). **b** Interclavicular ligament (white arrow indicating anterosuperior aspect) attached on sternum (transverse plane with caudal tilt). **c** Longitudinal view of sternal tendon of the sternocleidomastoid muscle (white arrows) covering the medial aspect of the sternoclavicular joint (slightly upward and outward oblique sagittal plane). White broken lines show the articular disc, thin white lines show the anterior sternoclavicular ligament. C, Clavicle; S, Sternum

In no cases could the anterior sternoclavicular ligament be differentiated from the underlying sternoclavicular capsule. This anterior capsuloligamentous complex was seen in each patient as a hypoechoic structure extending from the anterior edge of the clavicular notch of the manubrium sterni and from the adjacent first costal cartilage to the anterior and superior part of the sternal end of the clavicle. It was easily detected in an upward and outward oblique transverse plane (Fig. 3). Its mean thickness was 1.4 mm (SD = 0.2).

In each patient, the interclavicular ligament was seen as a hypoechoic fibrillar structure attaching to the superior aspect of the sternal end of each clavicle. It showed an anterosuperior concavity and was best seen on a transverse plane with 40–50° caudal tilt (Fig. 3). Its whole length could be seen using the linear probe but assessment at its clavicular and sternal insertions was best made using the hockey stick probe. Its clavicular end was applied closely against the sternoclavicular capsule. Its mean thickness was 1.3 mm (SD = 0.2). It inserted onto the jugular notch of the manubrium sterni in 36 patients (61% of cases), and at a distance from it in the other cases.

In no cases could the whole length of the costoclavicular ligament be clearly detected.

The sternal head of the sternocleidomastoid muscle was clearly seen as a hypoechoic structure attaching to the upper part of the anterior surface of the manubrium sterni, medially to the sternoclavicular joint and to the anterior sternoclavicular ligament insertion. In its downward and inward course, it partially coved the medial part of the sternoclavicular joint (Fig. 3). It was easily detected on a slightly upward and outward oblique sagittal plane (longitudinal view) or on a transverse plane.

Clavicular and sternal articular cartilages were not correctly seen in one joint (overweight patient). In all the other cases, only the superficial anterior aspect was seen, as an anechoic structure covering the subchondral bone, with a mean thickness of 0.5 mm (SD = 0.1).

**Fig. 4:**
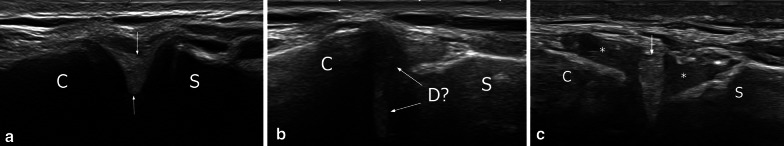
Intra-articular disc (upward and outward oblique transverse plane). **a** In this patient, the disc was clearly seen (arrows) as a hyperechoic triangular structure in the joint. **b** In this other patient with the arm alongside the body, the depiction of the disc was less clear. Moreover, intra-articular gas bubbles were associated (arrows). **c** With antepulsion of the shoulder, the joint fluid (*) and intra-articular disc (arrow) were displaced anteriorly, improving analysis of the disc. C: clavicle, S: sternum

**Fig. 5:**
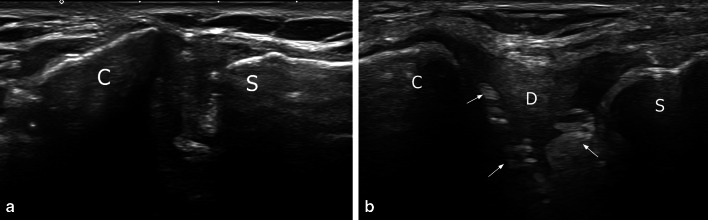
Intra-articular gas (arrows) preventing (**a**) or allowing (**b**) analysis of the intra-articular disc (upward and outward oblique transverse plane). C: clavicle, S: sternum

The articular disc was seen as an intraarticular hyperechoic triangular structure with its base on the anterior capsuloligamentous structure. It was clearly seen in 39 out of 59 joints (66.1% of cases) in patients with the arm alongside the body (Fig. 4). In 12 patients (20.3% of cases), its margins were not correctly analyzed with the arm alongside the body, but the disc was clearly seen under shoulder antepulsion (Fig. 4). In the other 8 patients (13.6% of cases), the articular disc could not be correctly analyzed, due to overweight (1 case), insufficient echogenicity (5 cases) or abundant intra-articular gas (2 cases) (Fig. 5). There was no calcification of the disc.

**Fig. 6:**
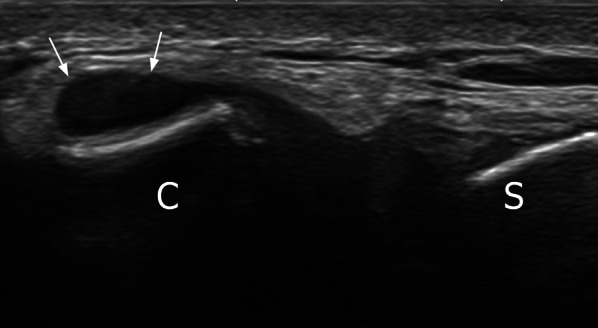
Joint effusion seen as a bulging synovial recess in front of the clavicular end (upward and outward oblique transverse plane). C: clavicle, S: sternum

Intra-articular joint gas (Fig. 5), seen as small round hyperechoic elements, mobile spontaneously or during movement, was seen in 20 patients (33.89% of cases). Joint effusion was seen, as a bulging synovial recess in front of the clavicular end, in 4 patients (6.8% of cases) (Fig. 6). There was no synovial hyperemia. The joint width showed a mean thickness of 7.5 mm (SD = 1.5).

Clavicular osteophytes were detected in 20 patients (33.9% of cases) (mean age, 42.7 years, SD = 13) and sternal osteophytes in 10 (16.9%) (mean age, 36 years, SD = 10). Irregularities of the bone surface at the anterior sternoclavicular ligament insertion area were seen in 7 patients (11.9%) at the clavicle (mean age, 39.8 years, SD = 9.8) and in 10 (16.9%) at the sternum (mean age, 36 years, SD = 11).

## Discussion

To the best of our knowledge, this is the first ultrasound study assessing the normal anatomy of the sternoclavicular joint. This is all the more surprising since this joint is superficial and therefore particularly accessible to this imaging modality. Understanding the anatomy is fundamental for a correct vision of the various components of the joint when using ultrasound. Our study showed that this technique can correctly analyze the anatomy of the anterior aspect of the sternoclavicular joint, while the posterior part is not accessible to ultrasound.

### Ligaments

The sternoclavicular joint is a saddle-shaped synovial joint. As the sternal end of the clavicle has a larger surface area than the adjacent sternum, the joint is reported to have little intrinsic stability [1, 11], stability rather depending primarily on surrounding sternoclavicular ligaments and the costoclavicular ligament, although which is the primary stabilizer is still debated [12–14].

The anterior sternoclavicular ligament was clearly seen both on dissection and in patients, as a broad oblique capsular thickening running from the anterosuperior aspect of the sternal end of the clavicle to the upper anterior edge of the manubrium sterni, with fibers inserting on the first costal cartilage. Differentiation between the ligament and underlying capsule was not possible in our study, either on dissection or on ultrasound, as previously reported for dissection in other studies [15, 16]. To the best of our knowledge, there are no data regarding the normal thickness of this structure in the anatomic or imaging literature. In the present study, mean thickness was 1.4 mm (SD = 0.2). This fact may have applications, particularly in the context of trauma or microtrauma, as the anterior sternoclavicular ligament is one of the most frequently injured ligaments in sprain or dislocation of the sternoclavicular joint [17, 18].

Data are sparse for interclavicular ligament anatomy. This structure was seen in each of our patients, but correct assessment may require an ultrahigh-frequency hockey stick probe and /or a large amount of gel due to the wavy outlines of the superior chest wall, particularly in thin patients. This ligament extended between the superior aspect of the sternal end of the clavicles, but our anatomic and ultrasound studies showed it could be attached to the upper manubrium sterni (in more than half patients on ultrasound). The clavicular end of the ligament could not be differentiated from the underlying articular capsule. This feature was previously reported on MRI [3]. This ligament is one of those involved in sternoclavicular joint stability [16]. However, to the best of our knowledge, the importance of manubrial insertion for its stabilizing effect has not been assessed.

The costoclavicular ligament extends from the undersurface of the medial clavicle to the superior rim of the first rib and costal cartilage. This ligament, which is not strictly speaking a component of the sternoclavicular joint, was not correctly seen on ultrasound in our study, probably because of its depth, as it is covered by the pectoralis major muscle. Elevation of the arm, which was not tried in this study, might improve its assessment by lifting the clavicle.

### Intra-articular structures

The clavicular cartilage has been reported to be thicker than the sternal cartilage [3, 19]. No difference in thickness was found in our study. However, only the superficial anterior aspect of the two cartilages was accessible to ultrasound, which probably limits the usefulness of this technique for cartilage assessment. The joint space could also be measured with ultrasound, but the clinical relevance of this remains to be demonstrated.

Sternoclavicular osteoarthritis is relatively common, although asymptomatic in most cases. Clavicular and sternal osteophytes were detected in 33.9% and 16.9% of our cases, respectively, despite the relatively young age of the population (median, 35 years). These rates are higher than in a recent CT-based study, which reported 89% sternoclavicular osteoarthritis in over-50-year-olds, in contrast to 9% in under-50 s [20]. The use of high-resolution ultrasound probes in our study might at least partly explain increased detection of small osteophytes. Also, bone irregularities at the anterior sternoclavicular ligament attachment were found in 11.9% of patients. These changes may be related to mechanical changes at the insertion site. As the joints assessed in the present study were asymptomatic, this means that osteophytes and entheseal bone irregularities can be seen in the absence of any clinical symptoms. However, further studies are needed to assess whether there could be a correlation between osteoarthritis-related changes of the joint and variations in thickness or echostructure of the capsular and/or ligamental structures, especially in symptomatic patients.

The intra-articular disc, which might function as an important shock absorber [21], lies between the joint surfaces of the clavicle and manubrium sterni, dividing the joint into two synovium-lined cavities [3, 16]. This fibrocartilaginous structure shows a robust insertion on the posterosuperior surface of the medial edge of the clavicle, with peripheral attachments to the anterior and posterior sternoclavicular capsuloligamentous structures [3, 16]. Inferiorly, it has a less robust attachment to the first costal cartilage and sternum. This nearly circular structure is thicker in its periphery and at its attachment sites, tapering centrally. The presence or absence of a central hole was not assessed in our study, because of the depth of this feature; only the anterior half of the disc was assessed with ultrasound, but was clearly seen as a hyperechoic triangular structure in most cases, either spontaneously with the arm alongside the body (66.1% of cases) or after shoulder antepulsion (20.3% of cases). The antepulsion maneuver was helpful, as it provided a narrowing of the sternoclavicular joint which pushed the joint fluid and intra-articular disc forward, improving analysis quality.

In the other cases, suboptimal disc analysis was due to overweight, poor echogenicity or abundant intra-articular gas bubbles hindering assessment. Degenerative changes in the disc with irregular margins can also explain poor visualization, despite the relatively young age of our population. It is well known that, with advancing age and development of osteoarthritis, there is progression from a complete and regular disc to an incomplete disc with a central hole and signs of degeneration and fraying [20]. Disc aspect in cadaver studies correlated with articular degenerative damage [15]. However, the clinical relevance of these changes is unclear. Chondrocalcinosis with deposition of calcium pyrophosphate dihydrate crystals can be seen in elderly patients [22], but no calcification of the disc was found in the present study.

Finally, joint effusion was seen, as a bulging synovial recess in front of the clavicular end, in a few cases (6.8%), without associated synovitis. Interestingly, these patients were athletic (bodybuilding, swimming); however, sports practice was not systematically assessed in our study.

### Limitations

We acknowledge several limitations in our study. Firstly, only 59 patients were included and the median age of 35 years was young. Larger studies might find different results for the detection prevalence and measurement of the anterior sternoclavicular structures. Secondly, intra- and inter-observer reproducibility was not assessed, as all the ultrasound examinations were analyzed consensually by two musculoskeletal radiologists; this may also have influenced detection prevalence. However, the main goal of the study was to describe the ultrasound aspect of these structures. Furthermore, we did not assess whether the appearance of these anatomical structures was symmetrical, which could be useful in clinical practice.

Finally, we did not perform several dynamic maneuvers to assess whether or not they could optimize the anatomical study of the sternoclavicular joint, especially for a better differentiation between anterior capsular and ligamental structures.

## Conclusion

In conclusion, our study demonstrated that the anterior sternoclavicular capsuloligamentous structure, interclavicular ligament and anterior intraarticular structures can be visualized on ultrasound. Potential applications must now be confirmed by clinical studies, particularly in patients with trauma and microtrauma.

## Data Availability

Data of this study are available from the corresponding author upon reasonable request.

## References

[1] Sewell MD, Al-Hadithy N, Le Leu A, Lambert SM (2013). Instability of the sternoclavicular joint: current concepts in classification, treatment and outcomes. Bone Joint J.

[2] Destouet JM, Gilula LA, Murphy WA, Sagal SS (1981). (1981) Computed tomography of the sternoclavicular joint and sternum. Radiology.

[3] Brossmann J, Stäbler A, Preidler KW, Trudell D, Resnick D (1996). Sternoclavicular joint: MR imaging–anatomic correlation. Radiology.

[4] Restrepo CS, Martinez S, Lemos DF (2009). Imaging appearances of the sternum and sternoclavicular joints. Radiographics.

[5] Tuscano D, Banerjee S, Terk MR (2009). Variations in normal sternoclavicular joints; a retrospective study to quantify SCJ asymmetry. Skeletal Radiol.

[6] Rodríguez-Henríquez P, Solano C, Peña A (2013). Sternoclavicular joint involvement in rheumatoid arthritis: clinical and ultrasound findings of a neglected joint. Arthritis Care Res.

[7] Wisniewski SJ, Smith J (2007). Synovitis of the sternoclavicular joint: the role of ultrasound. Am J Phys Med Rehabil.

[8] Umeda M, Kawashiri S-Y, Nishino A (2017). Synovitis of sternoclavicular and peripheral joints can be detected by ultrasound in patients with SAPHO syndrome. Mod Rheumatol.

[9] Kawashiri S, Edo Y, Kawakami A (2019). Early detection of inflammation and joint destruction revealed by ultrasound in a patient with sternoclavicular septic arthritis. Intern Med.

[10] Pourcho AM, Sellon JL, Smith J (2015). Sonographically guided sternoclavicular joint injection: description of technique and validation. J Ultrasound Med.

[11] Jazrawi L, Rokito A, Birdzell M, Zuckerman J (2001) Biomechanics of the shoulder, vol 2. Basic biomechanics of the musculoskeletal system, third ed. Lippincott Williams and Wilkins, Philadelphia (PA), pp 47–72

[12] Bearn JG (1967). Direct observations on the function of the capsule of the sternoclavicular joint in clavicular support. J Anat.

[13] Spencer EE, Kuhn JE, Huston LJ, Carpenter JE, Hughes RE (2002). Ligamentous restraints to anterior and posterior translation of the sternoclavicular joint. J Shoulder Elbow Surg.

[14] Tubbs RS, Shah NA, Sullivan BP (2009). The costoclavicular ligament revisited: a functional and anatomical study. Rom J Morphol Embryol.

[15] Van Tongel A, MacDonald P, Leiter J, Pouliart N, Peeler J (2012). A cadaveric study of the structural anatomy of the sternoclavicular joint. Clin Anat.

[16] Lee JT, Campbell KJ, Michalski MP (2014). Surgical anatomy of the sternoclavicular joint: a qualitative and quantitative anatomical study. J Bone Joint Surg.

[17] Allman FL (1967). Fractures and ligamentous injuries of the clavicle and its articulation. J Bone Joint Surg.

[18] Nettles JL, Linscheid RL (1968). Sternoclavicular dislocations. J Trauma.

[19] Dhawan R, Amol Singh R, Tins B, Hay SM (2018). Sternoclavicular joint. Shoulder Elbow.

[20] Lawrence CR, East B, Rashid A, Tytherleigh-Strong GM (2017). The prevalence of osteoarthritis of the sternoclavicular joint on computed tomography. J Elbow Shoulder Surg.

[21] Kiel J, Ponnarasu S, Kaiser K (2021) Sternoclavicular joint injury. In: StatPearls [Internet]. Treasure Island (FL): StatPearls Publishing 29939671

[22] Verhoeven F, Chouk M, Wendling D (2016). Ultrasonography: a useful tool for the diagnosis of chondrocalcinosis of the sternoclavicular joint. J Rheumatol.

